# END-OF-LIFE CARE DISPARITY FOR ASIAN AMERICAN PACIFIC ISLANDER AND WHITE AMERICANS WITH DEMENTIA

**DOI:** 10.1093/geroni/igae098.0379

**Published:** 2024-12-31

**Authors:** Hyosin Kim, Haiqun Lin, Anum Zafar, Bei Wu, Paul Duberstein, Olga Jarrín

**Affiliations:** Oregon State University, Corvallis, Oregon, United States; Rutgers, The State University of New Jersey, Newark, New Jersey, United States; Rutgers, The State University of New Jersey, New Brunswick, New Jersey, United States; New York University, New York, New York, United States; Rutgers, The State University of New Jersey, Piscataway, New Jersey, United States; Rutgers, The State University of New Jersey, New Brunswick, New Jersey, United States

## Abstract

Alzheimer’s disease and related dementias (ADRD) disproportionately affects Asian American and Pacific Islander (AAPI) older adults, similar to other minoritized groups. People living with ADRD have significant caregiving and end-of-life care needs. Little is known about racial disparities in end-of-life care for the AAPI Medicare beneficiaries with dementia. Using Medicare administrative, claims, and assessment from inpatient, hospice, and post-acute and long-term care settings for Medicare beneficiaries who died in 2019, differences in end-of-life care and place of death for AAPI (n= 20,301) and non-Hispanic White (n=762,443) Medicare beneficiaries with dementia and comparable levels of multimorbidity (median 6, IQR 5-8) were studied. Compared to White beneficiaries with dementia, AAPI beneficiaries with dementia were more likely to be dually enrolled in Medicare and Medicaid (60% vs. 32%), yet less likely to use home health care (52% vs. 55%) or nursing home care (54% vs. 64%) during the last 3 years of life; less likely to use hospice care during the last 6 months of life (52% vs. 65%); and more likely to die in an inpatient setting (34% vs. 26%) compared to their white counterparts. Differences were magnified among beneficiaries with high chronic disease burden (four or more comorbidities in addition to dementia). Underuse of hospice services and the high rates of Medicare-Medicaid dual enrollees among AAPI beneficiaries with dementia and multiple comorbidities warrant further attention, as the disease burden particularly increases with multiple chronic conditions.

